# Correction to “Long Noncoding RNA OVAAL Enhances Nucleotide Synthesis Through Pyruvate Carboxylase to Promote 5‐Fluorouracil Resistance in Gastric Cancer”

**DOI:** 10.1111/cas.70529

**Published:** 2026-09-05

J.‐N., Tan , S.‐N. Zhou , and W. Zhang , et al. “Long Noncoding RNA OVAAL Enhances Nucleotide Synthesis Through Pyruvate Carboxylase to Promote 5‐Fluorouracil Resistance in Gastric Cancer,” Cancer Science 113, (2022): 3055‐3070, 10.1111/cas.15453.35657686 PMC9459305

In the above article, Figure 2N was incorrect. The updated figure is shown below:

**Figure 2N**

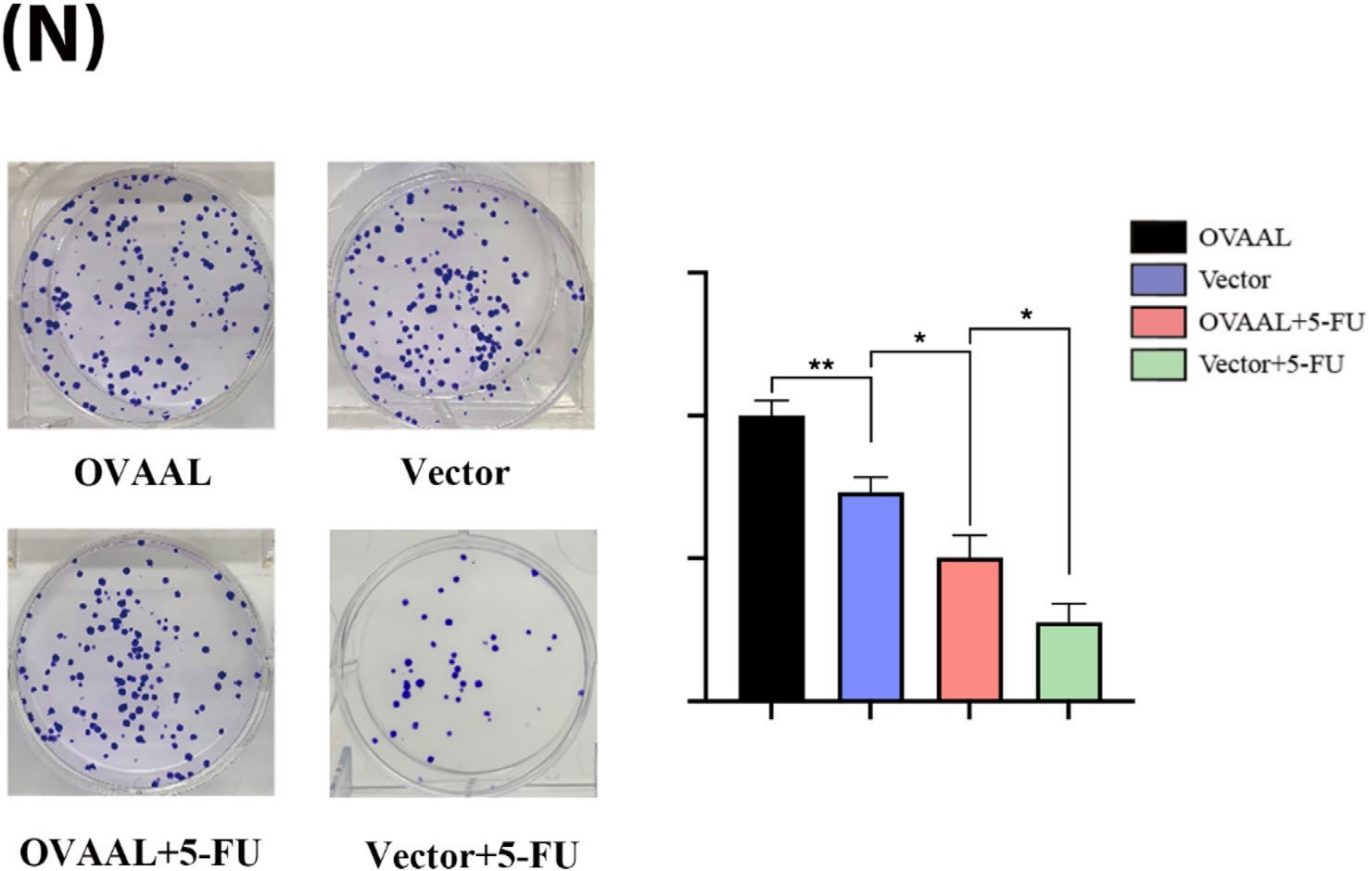

We apologize for the error.

